# *Cis* interaction between sialylated FcγRIIA and the αI-domain of Mac-1 limits antibody-mediated neutrophil recruitment

**DOI:** 10.1038/s41467-018-07506-1

**Published:** 2018-11-29

**Authors:** Gurpanna Saggu, Koshu Okubo, Yunfeng Chen, Ravi Vattepu, Naotake Tsuboi, Florencia Rosetti, Xavier Cullere, Nathaniel Washburn, Suhail Tahir, Aaron M. Rosado, Steven M. Holland, Robert M. Anthony, Mehmet Sen, Cheng Zhu, Tanya N. Mayadas

**Affiliations:** 1 Department of Pathology, Brigham and Women’s Hospital & Harvard Medical School, Boston, MA 02115 USA; 2 Institute for Bioengineering and Biosciences, Georgia Institute of Technology, Atlanta, GA 30332 USA; 3 Division of Rheumatology, Allergy and Immunology, Massachusetts General Hospital & Harvard Medical School, Charlestown, MA 02129 USA; 4 Momenta Pharmaceuticals, Cambridge, MA 02142 USA; 5 National Institute of Allergy and Infectious Diseases, Laboratory of Clinical Infectious Diseases, National Institutes of Health, Bethesda, MD 20814 USA; 6 Department of Biology and Biochemistry, University of Houston, Houston, TX 77204 USA; 7 Present Address: Department of Nephrology, Fujita Health University School of Medicine, Toyoake, Aichi 470-1192 Japan; 8 Present Address: Department of Immunology and Rheumatology, Instituto Nacional de Ciencias Médicas y Nutrición Salvador Zubirán, Mexico City, 14080 Mexico

## Abstract

Vascular-deposited IgG immune complexes promote neutrophil recruitment, but how this process is regulated is still unclear. Here we show that the CD18 integrin Mac-1, in its bent state, interacts with the IgG receptor FcγRIIA in *cis* to reduce the affinity of FcγRIIA for IgG and inhibit FcγRIIA-mediated neutrophil recruitment under flow. The Mac-1 rs1143679 lupus-risk variant reverses Mac-1 inhibition of FcγRIIA, as does a Mac-1 ligand and a mutation in Mac-1’s ligand binding αI-domain. Sialylated complex glycans on FcγRIIA interact with the αI-domain via divalent cations, and this interaction is required for FcγRIIA inhibition by Mac-1. Human neutrophils deficient in CD18 integrins exhibit augmented FcγRIIA-dependent recruitment to IgG-coated endothelium. In mice, CD18 integrins on neutrophils dampen IgG-mediated neutrophil accumulation in the kidney. In summary, *cis* interaction between sialylated FcγRIIA and the αI-domain of Mac-1 alters the threshold for IgG-mediated neutrophil recruitment. A disruption of this interaction may increase neutrophil influx in autoimmune diseases.

## Abstract (web summary)

Deposited immune complexes (IC) promote neutrophil recruitment, but the fine tuning of this process is still unclear. Here the authors show that the *cis* interaction of the IC receptor, FcγRIIA and CD18 integrin, Mac-1, on the neutrophil surface modulates neutrophil adhesion, with FcγRIIA sialylation specifically implicated in this interaction.

## Introduction

FcγRIIA, a uniquely human ITAM-containing receptor, is a key activating receptor for complexed IgG present on multiple myeloid cells and platelets^1,2^. For example, FcγRIIA on dendritic cells is essential for generating anti-tumor T cell responses^1^ whereas on neutrophils promotes cytotoxic functions^2^ and on macrophages facilitates immune complex (IC) clearance^2^. Moreover, studies have identified a key role for FcγRIIA, and other low affinity FcγRs, in neutrophil and monocyte capture by IgG-immune complexes deposited on the endothelium in vitro and in vivo^3–8^. FcγRIIA also positively regulates Toll-like (TLRs)^9,10^ and cytokine receptors^10^ and FcγRIIA SNPs associate with a range of diseases from SLE^11^ and rheumatoid arthritis^12^ to Kawasaki disease^13,14^. Thus, determining how FcγRIIA activity is regulated may have broad significance for disease pathogenesis. Here, we studied the mechanisms that regulate FcγRIIA mediated neutrophil recruitment to deposited immune complexes, potentially one of the earliest steps of inflammation and subsequent tissue damage in autoimmune disease^15^.

The CD18 integrins, composed of a common CD18 (β2) subunit complexed with unique CD11 (α) subunits (CD11a–d), are a major family of adhesion molecules on myeloid cells. CD18 integrin binding to ligand relies on allosteric changes transmitted to and from the ligand binding αI-domain, which has a divalent cation in the metal ion dependent adhesion site (MIDAS) that coordinates acidic residues in the ligand. Allostery relay is triggered by inflammatory mediators or heterologous receptors, which generate intracellular signals that impinge on the cytoplasmic tail of the CD18 subunit. These signals shift the integrin from a bent/closed to various degrees of active/open, extended conformations that have increased ligand binding affinity^16,17^. Historically, CD18 integrins have been considered to be pro-inflammatory. Mac-1 (CD11b/CD18) and LFA-1 (CD11a/CD18) promote neutrophil recruitment with an absence of all CD18 integrins in Leukocyte adhesion deficiency I (LADI) patients leading to recurrent infections^18^. Mac-1 also contributes to sustained inflammation and tissue damage and enhances the function of heterologous receptors such as Toll-like receptors (TLRs), FcγRs and the urokinase receptor (uPAR) by regulating intracellular signaling^19^. However, Mac-1 can also inhibit TLR function^20–22^. A similar duality in Mac-1 function may exist for FcγRs. Mac-1 promotes FcγRs mediated neutrophil adhesion and cytolysis of IgG-opsonized targets^23–26^ and sustains neutrophil adhesion and injury in glomerulonephritis (GN) induced by in situ deposition of anti-glomerular basement membrane (GBM) antibody^25,27^. On the other hand, in a mouse model of GN induced by the passive transfer of IgG/ICs-containing systemic lupus erythematosus (SLE) sera, FcγRIIA mediated glomerular neutrophil accumulation occurs only when Mac-1 is absent^28^. The inhibitory role of Mac-1 may have relevance in humans as the non-synonymous Mac-1 functional variant rs1143679 (Arg to His substitution at position 77 in the extracellular β-propeller domain of the CD11b subunit) that increases risk for lupus nephritis decreases Mac-1 ligand binding^19,29^ by interfering with allostery relay to the ligand binding αI-domain^30^.

Although much is known about FcγRIIA effector functions^1,2^, how the ligand binding activity of this receptor is regulated remains less understood. Human neutrophils also express the GPI-linked low affinity receptor, FcγRIIIB^2^ but the binding kinetics of FcγRIIA are not affected by FcγRIIIB^31^. The ratio of activating FcγRs and the inhibitory FcγRIIB sets the threshold for FcγR activation and subsequent responsiveness of immune cells to ICs^1^ but FcγRIIB is low to undetectable in neutrophils and monocytes of most individuals^32^. Recent studies show that FcγR-IgG interactions are regulated not only by the IgG subclass composition but also by the glycan structure of the Fc region wherein IgG sialylation reduces FcγR affinity^1^. Notably, engineered sialylation with soluble glycosyltransferases in vivo was recently shown to reduce inflammation and tissue injury in models of arthritis and nephrotoxic nephritis^33^.

Here, we test the hypothesis that an ectodomain *cis*-interaction between FcγRIIA and bent Mac-1 represents a mechanism for regulating FcγRIIA affinity for IgG and subsequent immune complex-mediated neutrophil recruitment. We show that a divalent cation-dependent *cis* interaction between the α5-helix of the Mac-1 ligand binding αI-domain, E^253^-R^261^, and the heavily sialylated ectodomain of FcγRIIA reduces FcγRIIA affinity for IgG and subsequent neutrophil recruitment to deposited ICs under physiological flow conditions. Using neutrophils from patients with LAD1 and a mouse model of acute anti-GBM nephritis with neutrophil selective changes in CD18 expression, we show that FcγR-mediated neutrophil recruitment is negatively regulated by CD18 integrins. Thus, modulation of FcγRIIA sialylation and Mac-1 allostery may represent a mechanism for fine tuning FcγRIIA responses to IgG immune complexes in myeloid cells.

## Results

### CD18 integrins limit FcγRIIA-immune complex interactions

**Fig. 1:**
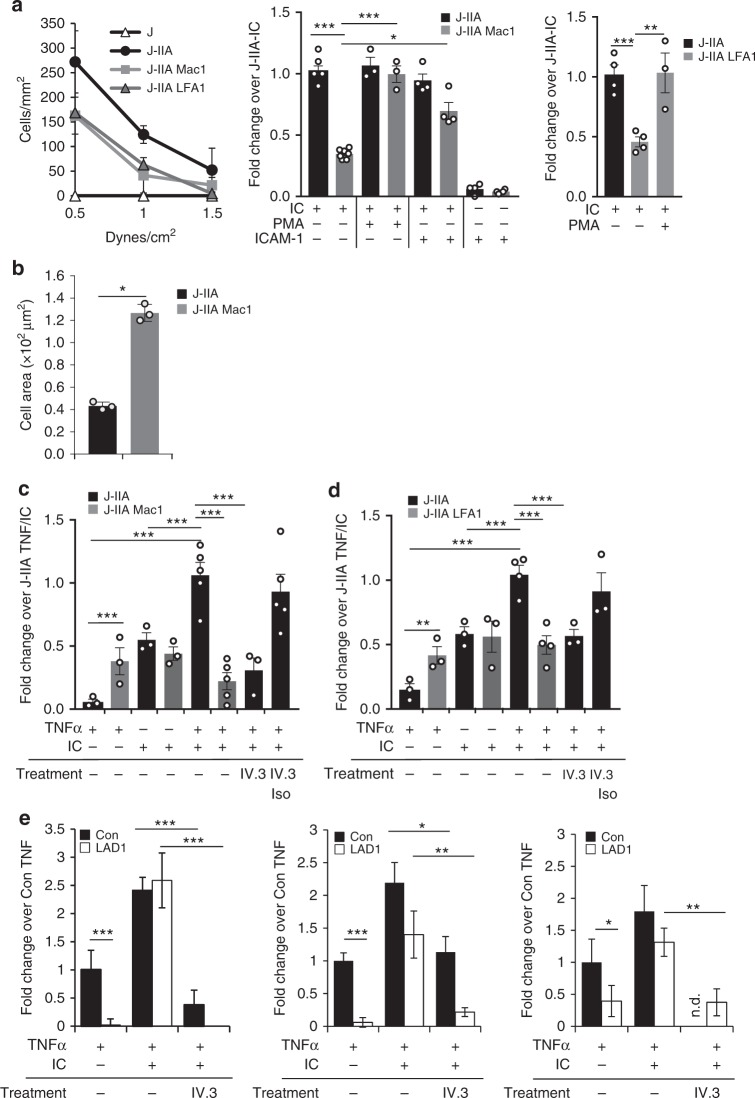
CD18 integrins, Mac-1 and LFA-1 in *cis* inhibit FcγRIIA mediated interaction with ICs under flow. Cells were perfused under physiological flow conditions over plate immobilized ICs (**a**, **b**) or TNF activated human dermal microvascular endothelial cells (HDMEC) with or without ICs generated by incubating cells with mouse anti-endoglin mAb (anti CD105) and rabbit anti-mouse IgG (**c**, **d**). The number of adherent cells was assessed and averaged. **a** Jurkat cells expressing FcγRIIA (J-IIA) with Mac-1 (J-IIA Mac1) or with LFA-1 (J-IIA LFA1) were perfused over immobilized ICs or ICs co-immobilized with ICAM with or without prior PMA treatment. Left panel shows cell adhesion to immobilized ICs at the indicated shear stress. Data is presented as average ± SD of one representative of four experiments with duplicate coverslips per condition. Right panel, bar graphs represent fold change compared to J-IIA cells at 1.0 dyne/cm^2^ under indicated conditions. Data is presented as average ± SEM. **b** After perfusing cells over immobilized ICs, the coverslips were fixed, permeabilized, and stained with rhodamine-phalloidin to visualize the actin cytoskeleton. Bar graphs represent cell area. Data is presented as average ± SEM of *n* = 3. **c**, **d** Indicated Jurkat cells were perfused over TNFα-activated HDMEC with or without deposited ICs. The number of adherent cells was assessed. No rolling was observed. Cells were pre-treated where indicated with anti-FcγRIIA (IV.3) or isotype controls for 30 min at 37 °C. Bar graphs represent fold change compared to J-IIA TNF/IC. Data is presented as average ± SEM of *n* = 3–5. **e** Isolated human peripheral blood PMNs from 3 normal donors (control) and 3 LAD1 patients were pre-treated where indicated with anti-FcγRIIA (IV.3) and perfused across TNFα activated HDMEC with or without IC coating and analyzed as in **c**. Results for each of the three LAD1 patients with 15.3, 17.9, and 22.7% CD18 compared to their respective healthy donors (Con) is shown. Data is average fold change compared to TNF alone control ± SD from duplicate coverslips. For **a**, **b** *p < 0.05; ***p < 0.001 using the Student’s unpaired *t*-test. For **c**–**e** *p < 0.05; ***p < 0.001 using the one way ANOVA followed by Sidak’s Multiple comparison test

To examine Mac-1 effects on FcγRIIA function, we generated stable Jurkat T cell lines, previously mutagenized to lack CD18 integrin LFA-1 (J)^34^, that express human FcγRIIA in the absence (J-IIA) or presence of human Mac-1 (J-IIA Mac1). We also expressed human FcγRIIA on Jurkat cells with intact endogenous LFA-1 expression (J-IIA LFA1). The ratio of FcγRIIA to Mac-1 and LFA-1 was higher in the J-IIA Mac1 and J-IIA LFA1 than in healthy, normal human and mouse neutrophils, so the impact of Mac-1 and LFA-1 on FcγRIIA may be underestimated in these cells (Supplementary Figure 1A). FcγRIIA expression was similar across all cell lines (Supplementary Figure 1B). We evaluated adhesion of the Jurkat cell lines to plate-immobilized ICs under physiological shear flow conditions. J-IIA but not J cells robustly bound immobilized ICs, which was significantly reduced in J-IIA Mac-1 cells. A comparable decrease was observed in J-IIA LFA1 cells. The reduction in binding of J-IIA Mac-1 and J-IIA LFA1 cells was rescued by treatment of cells with a phorbol ester (PMA), a known potent activator of PKC and CD18 integrins^35^ (Fig. 1a), while PMA had no effect on J-IIA binding (Fig. 1a). Moreover, binding of J-IIA Mac-1 was partially rescued when ICs were co-immobilized with the CD18 integrin ligand ICAM-1 (Fig. 1a). Notably, J-IIA Mac-1 cells did not adhere to ICAM-1 alone (Fig. 1a) likely because of the need for IC engagement of FcγR to activate Mac-1^36^ to bind ICAM-1. Notwithstanding the reduction in binding of J-IIA Mac1 cells, the cells that bound exhibited increased spreading on ICs compared to J-IIA cells (Fig. 1b), as expected from previous studies^25,37^.

Next, we assessed the effect of Mac-1 and LFA-1 on FcγRIIA mediated binding to TNF stimulated human dermal microvascular endothelial cells (HDMEC) coated with anti-endoglin^5,6^, which models anti-endothelial cell antibody (AECA) deposition observed in autoimmune patients. AECA enhances neutrophil adhesion to the TNF activated endothelium (TNF/AECA) under flow that is FcγRIIA dependent^5,6^. In the Jurkat cell lines, J-IIA cells were recruited only when ICs were present and this was inhibited by a FcγRIIA blocking antibody. J-IIA Mac1 cells exhibited greater binding to TNF treated endothelial cells compared to J-IIA that is attributed to Mac-1 binding to ICAM-1 on the endothelium. In contrast, J-IIA Mac1 binding to TNF/AECA was significantly reduced compared to J-IIA (Fig. 1c). A similar result was obtained with J-IIA LFA1 (Fig. 1d). On the other hand, FcγRIIA had no effect on Mac-1 and LFA-1 mediated cell adhesion to TNF activated endothelium (Supplementary Figure 1C) suggesting that although Mac-1 inhibits FcγRIIA activity, the converse does not occur. To evaluate the relevance of our findings in human neutrophils, we compared the binding of neutrophils from normal human donors to TNF activated and/or TNF/AECA coated HDMECs with neutrophils from three patients with Leukocyte Adhesion Deficiency 1 (LAD1)^38^, which had equivalent surface levels of FcγRIIA and FcγRIIIB but reduced CD18 integrins compared to normal neutrophils (Supplementary Figure 1D). Notably, although CD11b biosynthesis is not dependent on CD18, an unassociated CD11b has improper topology due to incomplete folding of its β-propeller domain^39^ and is thus most likely to be incompetent for ligand binding. Normal human neutrophils bound to TNF activated HDMEC while LAD1 neutrophils rolled but did not adhere (Fig. 1e) as expected^38^. Normal human neutrophils exhibited an increase in binding to TNF/AECA endothelial cells, which was inhibited by a functional blocking anti-FcγRIIA antibody (Fig. 1e), as previously reported^5,6^. LAD1 neutrophils robustly bound TNF/AECA endothelial cells despite the low to absent binding to endothelium activated with TNF alone and this was completely FcγRIIA dependent (Fig. 1e). The results with LAD1 neutrophils is in seeming contradiction to the reported inhibition of neutrophil binding to TNF/AECA endothelial cells when anti-CD18 integrin antibody TS1/18 is added^5^. Importantly, this report also showed that ICAM-1, the chemokine IL-8, released by the TNF activated endothelium and its receptor CXCR1/2 on the neutrophil^5^, which induces integrin activation by inside-out signaling^40^ are also required for neutrophil binding to TNF/AECA endothelial cells. A model depicted in Supplementary Figure 2, reconciles both sets of results: In normal neutrophils flowed across TNF/AECA endothelial cells, IL-8/CXCR1-mediated integrin activation leads to integrin binding to ICAM-1 *in trans*, which releases the inhibition of FcγRIIA. TS1/18 antibody prevents integrin extension and *trans* interaction with ICAM-1 thus resulting in continued inhibition of FcγRIIA. AECA coating on unactivated endothelial cells fails to support neutrophil adhesion^5,6^ due to the absence of IL-8/CXCR1 for integrin activation, low surface ICAM-1 for integrin binding, and an absence of mediators that may upregulate FcγRIIA activity^41,42^. LAD1 neutrophils fails to bind to ICAM-1 on TNF activated endothelial cells as this requires the CD18 integrins. However, they robustly bind TNF/AECA endothelial cells because CD18 deficiency leads to a release of FcγRIIA for binding to IgG-ICs. CD18 integrin inhibition of FcγRIIA is more dramatic in J-IIA Mac-1 and J-IIA LFA-1 cell lines (Fig. 1d) than in neutrophils as Jurkats cells, which are an immortalized line of human T cells, lack CXCR1/2 and therefore cannot respond to IL-8 to activate integrins in the context of the TNF activated endothelium.

### Mac-1 αI domain lowers FcγRIIA affinity for IgG

**Fig. 2:**
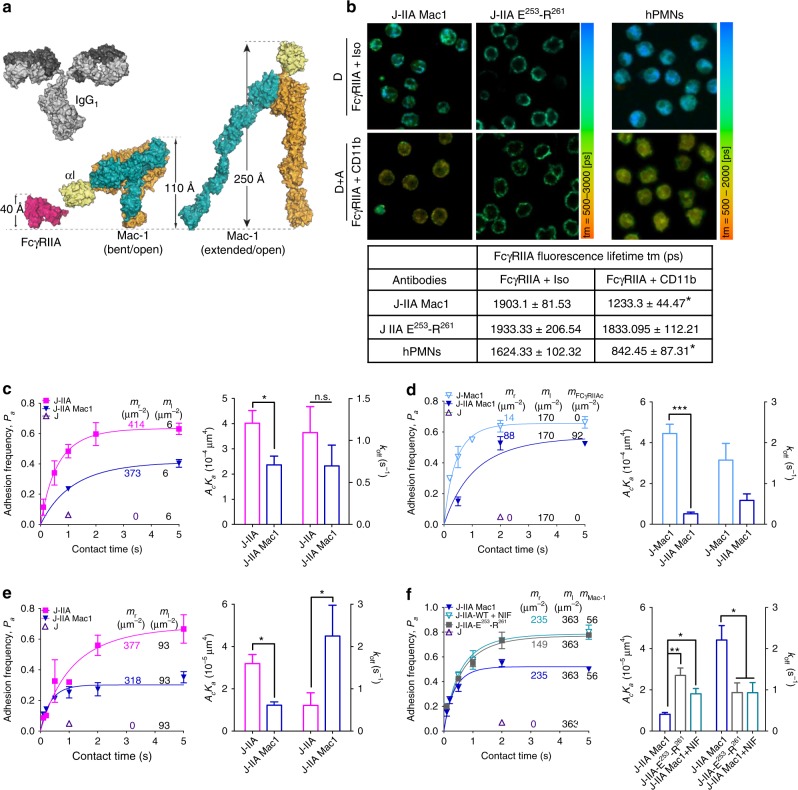
Mac-1’s αI domain interacts in *cis* with FcγRIIA’s ectodomain to lower FcγRIIA’s affinity for IgG. **a** Model of FcγRIIA, IgG, and Mac-1 in bent/closed and extended/open conformation based on published crystal structures. **b** FLIM was conducted on Jurkat cells expressing Mac-1 and FcγRIIA by staining with the donor (D) antibody for anti-FcγRIIA (IV.3 AF488) and the acceptor (A) antibody for Mac-1 (ICRF44 AF568) or an isotype control. FLIM images were taken and presented in pseudocolors from red to green. The fluorescence lifetimes of the donor in the absence (D) or presence (A + D) of the acceptor antibody were calculated. *n* = 3 experiments. *p < 0.01; unpaired *t*-test. **c**–**f** BFP measurements of adhesion frequency-versus-contact time of the indicated Jurkat cells (J-IIA) alone or with wild-type Mac-1 (WT), or Mac-1 lacking E^253^-R^261^ (E MT) with or without NIF treatment against bead immobilized Mac-1. **c** bead immobilized FcγRIIA (**d**) or bead immobilized IgG (**e**, **f**). The absence or presence of binding after a contact of given duration was analyzed to obtain the adhesion frequency. Bar graphs show effective 2D affinity (A_c_K_a_, E) and off-rate (k_off_, F) for each cell line. The adhesion frequencies of J-IIA-WT were lower than those of J-IIA, J-IIA-E MT, and NIF treatment of J-IIA-WT increased the effective FcγRIIA 2D affinity. Data is average ± SD. *p < 0.05, **p < 0.01, ***p < 0.001, by unpaired, two-tailed Student’s *t*-test

Our analysis of the crystal structures of CD18 integrins^43,44^, FcγRIIA^45^ and IgG^46^ combined with a report that Mac-1 αI-domain can interact with FcγRIIA ectodomain^37^ led us to propose a spatial model wherein Mac-1 in its bent/open conformation interacts with FcγRIIA laterally via its ligand binding αI-domain to curtail *trans*-interaction of FcγRIIA with IgG-ICs (Fig. 2a). Fluorescence lifetime imaging microscopy (FLIM) analysis of J-IIA Mac1 cells, using antibodies to the ligand-binding region of FcγRIIA and to the Mac-1 ligand binding αI-domain, suggested a close proximity between these two regions on the cell surface of J-IIA Mac-1 cells and human neutrophils (Fig. 2b). Next, direct molecular interactions were examined using a biomembrane force probe (BFP). We measured the adhesion frequency versus contact time between J-IIA and J-IIA Mac1 cells and beads immobilized with recombinant Mac-1 presented in *trans*. J-IIA cells interacted with Mac-1 in *trans* and this interaction was weakened when Mac-1 was present on the cell surface alongside FcγRIIA in *cis*, as reflected by the significantly lowered effective affinity (*A*_c_*K*_a_) (Fig. 2c). Note that this lower *A*_c_*K*_a_ is an averaged value over all the FcγRIIA copies on the cell surface; this included the ones engaged by *cis* Mac-1 and therefore adopting low affinity for *trans* Mac-1 binding, and the others free from *cis* Mac-1 interaction whose affinity for *trans* Mac-1 should remain unchanged. Also, using J-Mac1 and J-IIA Mac1 cells and bead-immobilized FcγRIIA we observed that Mac-1 interacted with FcγRIIA in *trans* and this interaction was reduced when FcγRIIA was present with Mac-1 in *cis* (Fig. 2d). These data suggest that Mac-1 and FcγRIIA directly interact in *cis*, which hinders their respective *trans* interaction to their counterparts on an opposing surface. To examine whether Mac-1 reduces FcγRIIA affinity for ligand, we used J-IIA cells and J-IIA Mac-1 cells presented with IgG-coated beads. We found that the *A*_c_*K*_a_ of J-IIA Mac-1 cells binding to the IgG-coated beads was significantly lower compared to J-IIA cells (Fig. 2e). In addition, the IgG off-rate was higher for J-IIA-WT compared to J-IIA cells suggesting that Mac-1 may be able to compete with established FcγRIIA-IgG binding (Fig. 2e). To assess the role of Mac-1’s ligand binding αI domain in the observed inhibition of FcγRIIA we used two approaches. First, we treated the J-IIA Mac1 cells with neutrophil inhibitory factory (NIF), a protein from hookworm that binds to the Mac1 αI-domain^47,48^ at E^253^-R^261^ ^49^. Second, we generated J-IIA cells expressing Mac-1 mutated in the E^253^-R^261^ region of the αI-domain that had surface levels of FcγRIIA and Mac-1 that were comparable to J-IIA Mac-1 cells (Supplementary Figure 1B). Both J-IIA Mac1 cells treated with NIF and JIIA cells expressing the Mac-1 E^253^-R^261^ mutant failed to reduce FcγRIIA affinity for bead immobilized IgG (Fig. 2f). Thus, the αI-domain of Mac-1 interacts with the ectodomain of FcγRIIA through its E^253^-R^261^ NIF binding region to reduce the affinity of FcγRIIA for IgG. Accordingly, the proximity of Mac-1 E^253^-R^261^ to FcγRIIA was reduced compared to wild-type Mac-1 as assessed by FLIM (Fig. 2b).

**Fig. 3:**
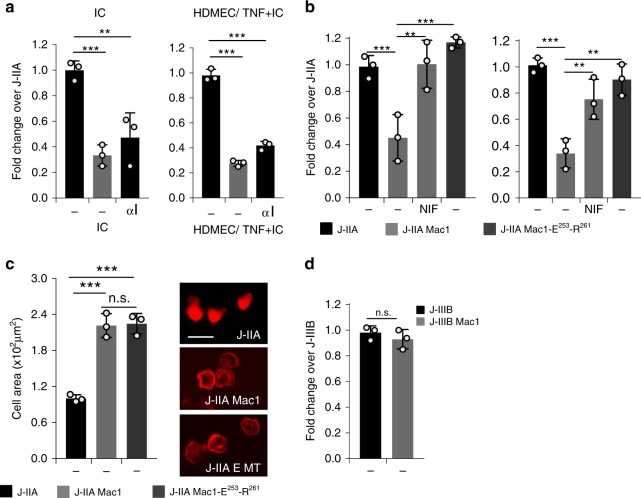
Mac-1’s αI-domain reduces FcγRIIA binding to ICs, but does not affect cell spreading or FcγRIIIB binding to ICs. Jurkat cells expressing either FcγRIIA or FcγRIIA and Mac-1 with or without pretreatment with recombinant αI-domain (100 μg/ml) for 30 min at 37 °C (**a**), without or with pretreatment with recombinant NIF (10 nM) (**b**), or J-IIA cells expressing Mac-1-E^253^-R^261^ mutant (**b**) were perfused at 1 dyne/cm^2^ over IC-coated coverslips (**a** and **b**, left panels) or TNF activated and IC coated HDMEC on coverslips (**a** and **b**, right panels). The number of adherent Jurkats was assessed and averaged. Data are represented as fold change compared to J-IIA cells of an average of 3 experiments ± SEM. **c** Indicated Jurkat cell lines were seeded onto immobilized ICs on coverslips for 30 min under static conditions. Coverslips were fixed, permeabilized, and stained with Alexa 568 phalloidin to visualize the actin cytoskeleton. Bar graphs represent average adherent cell area and representative images are shown. Data is presented as average ± SEM of *n* = 3. **d** Jurkat cells expressing FcγRIIIB without (J-IIIB) or with Mac-1 (J-IIIB Mac1) were perfused over coverslip immobilized ICs at 1 dyne/cm^2^ and the number of adherent cells were determined. Data are represented as fold change compared to J-IIIB. Data is average of 3 experiments ± SEM **p < 0.01, ***p < 0.001 using the One way ANOVA followed by Sidak’s Multiple comparison test

Next, we show that the interaction of Mac-1 αI-domain with FcγRIIA has functional consequences. J-IIA cells pretreated with recombinant, soluble αI-domain of Mac-1 exhibited a reduction in binding to immobilized ICs and to TNF/AECA endothelial cells under flow (Fig. 3a). Treatment of J-IIA Mac-1 cells with NIF reversed Mac-1 inhibition of FcγRIIA in both flow assays and the inhibitory effect of Mac-1 was also abolished in the E^253^-R^261^ mutant (Fig. 3b). Importantly, the E^253^-R^261^ mutant had no effect on FcγRIIA mediated cell spreading on ICs (Fig. 3c). In contrast to the inhibitory role of Mac-1 on FcγRIIA, Mac-1 had no effect on human FcγRIIIB interaction with ICs as assessed in flow assays using Jurkat cell lines engineered to stably express human FcγRIIIB in the absence (J-IIIB) or presence (J-IIIB Mac1) of Mac-1 (Fig. 3d). As Mac-1 has been shown to inhibit TLR4 function^19,50^, we also tested whether Mac-1 interacts with TLR4 in *cis*. FLIM analysis revealed that these two receptors were not in close proximity on the surface of human neutrophils (Supplementary Figure 3).

In summary, the interaction of E^253^-R^261^ in the αI-domain of Mac-1 with FcγRIIA’s ectodomain curtails FcγRIIA’s affinity for ICs, and therefore cell capture under flow, an inhibition that is reversed by Mac-1 activation and Mac-1 ligand (NIF) binding.

### Mac-1 R77H variant fails to interact with FcγRIIA in *cis*

**Fig. 4:**
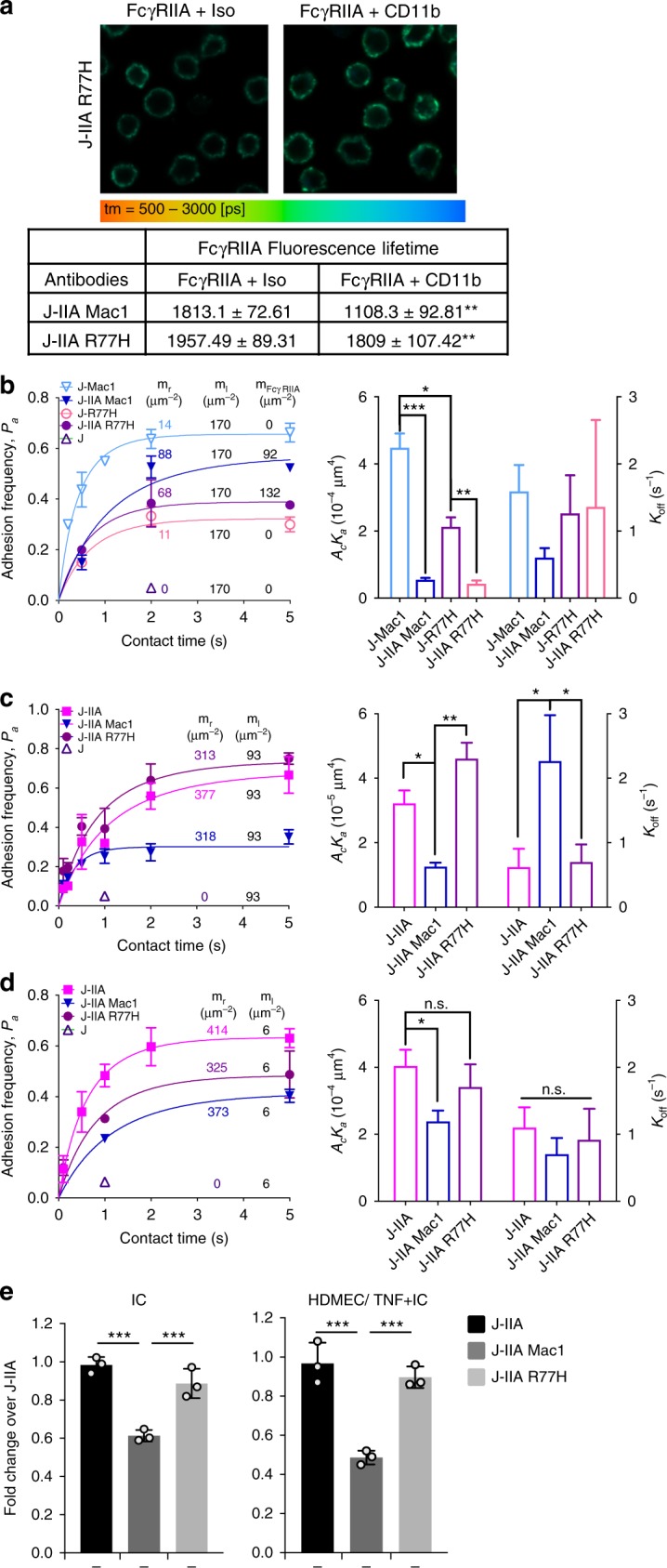
The lupus variant, Mac-1 R77H does not interact with FcγRIIA in *cis*, and fails to reduce FcγRIIA affinity for IgG and binding to ICs under flow. **a** FLIM was conducted on Jurkat cells expressing Mac-1 WT or R77H variant and FcγRIIA by staining with the donor (D) antibody for anti-FcγRIIA and the acceptor (A) antibody for Mac-1 or isotype control and the fluorescence lifetimes were calculated as described in Fig. 2. *n* = 3 experiments. **p < 0.01; unpaired *t*-test. **b**–**d** BFP measurements of adhesion frequency-versus-contact time (left panels) and kinetic parameters (right panels) of the indicated Jurkat cells (J-IIA) alone or with wild-type Mac-1 (J-IIA Mac1), or Mac-1 R77H variant (J-IIA-R77H) against beads immobilized FcγRIIA (**b**), IgG (**c**), or Mac-1 (**d**). Data is presented as mean ± SD. *p < 0.05, **p < 0.01, ***p < 0.001, by unpaired, two-tailed Student’s *t*-test. **e** Indicated Jurkat cells were perfused at 1 dyne/cm^2^ over IC-coated glass coverslips (left) or TNF activated and IC coated HDMEC plated coverslips (right). The number of adherent Jurkats was assessed and averaged. Data is represented as fold change compared to J-IIA cells at 1 dyne/cm^2^ of an average of 4 experiments ± SEM. ***p < 0.001, using the One way ANOVA followed by Sidak’s Multiple comparison test

The Mac-1 R77H functional variant resides in the β-propeller domain outside of the αI-ligand binding domain^19,51^. Moreover, R77H reduces 2D ligand-binding affinity and force-regulated dissociation of receptor-ligand bonds by perturbing allostery relay from the β-propeller in which it resides to the ligand binding αI-domain^30^. The R77H phenotype could be rescued by forcing the tail and lower leg separation of the α and β subunits in the absence of constitutive integrin extension and headpiece opening^30^. Given these data, we considered the possibility that the interaction of Mac-1 with FcγRIIA, which we propose serves as a “*cis* ligand”, will be similarly perturbed by R77H due to defects in allostery relay to the αI-domain of Mac-1. To address this hypothesis, we generated a stable Jurkat cell line expressing the R77H Mac-1 variant and FcγRIIA with surface levels of FcγRIIA and Mac-1 similar to J-IIA Mac-1 cells (Supplementary Figure 1B). FLIM analysis revealed that the αI-domain of the R77H variant of Mac-1 did not remain in close proximity to FcγRIIA compared to WT Mac-1 (Fig. 4a). In addition, in a BFP assay that assesses direct binding of cells expressing either WT (J-IIA Mac-1) or R77H (J-IIA R77H) Mac-1 to beads coated with recombinant FcγRIIA, we found that R77H significantly reduced the effective affinity to FcγRIIA as compared to WT Mac-1 (Fig. 4b). Accordingly, using BFP measurements of J-IIA cells expressing either WT or R77H Mac-1 against beads coated with IgG, we found that the R77H mutant had a reduced capacity to inhibit FcγRIIA binding to IgG (Fig. 4c). Conversely, when recombinant Mac-1 was immobilized on the bead and the interaction of IIA to Mac-1 in *trans* was measured by BFP, we found that R77H Mac-1 in *cis* could not inhibit the FcγRIIA-Mac-1 *trans* interaction (Fig. 4d). Thus, R77H perturbs Mac-1-FcγRIIA interaction and the subsequent FcγRIIA 2D affinity for IgG. Consistent with these findings, R77H failed to inhibit FcγRIIA mediated tethering to immobilized ICs or TNF/AECA coated HDMECs under flow (Fig. 4e).

### Mac-1 αI-domain interacts with sialylated FcγRIIA

**Fig. 5:**
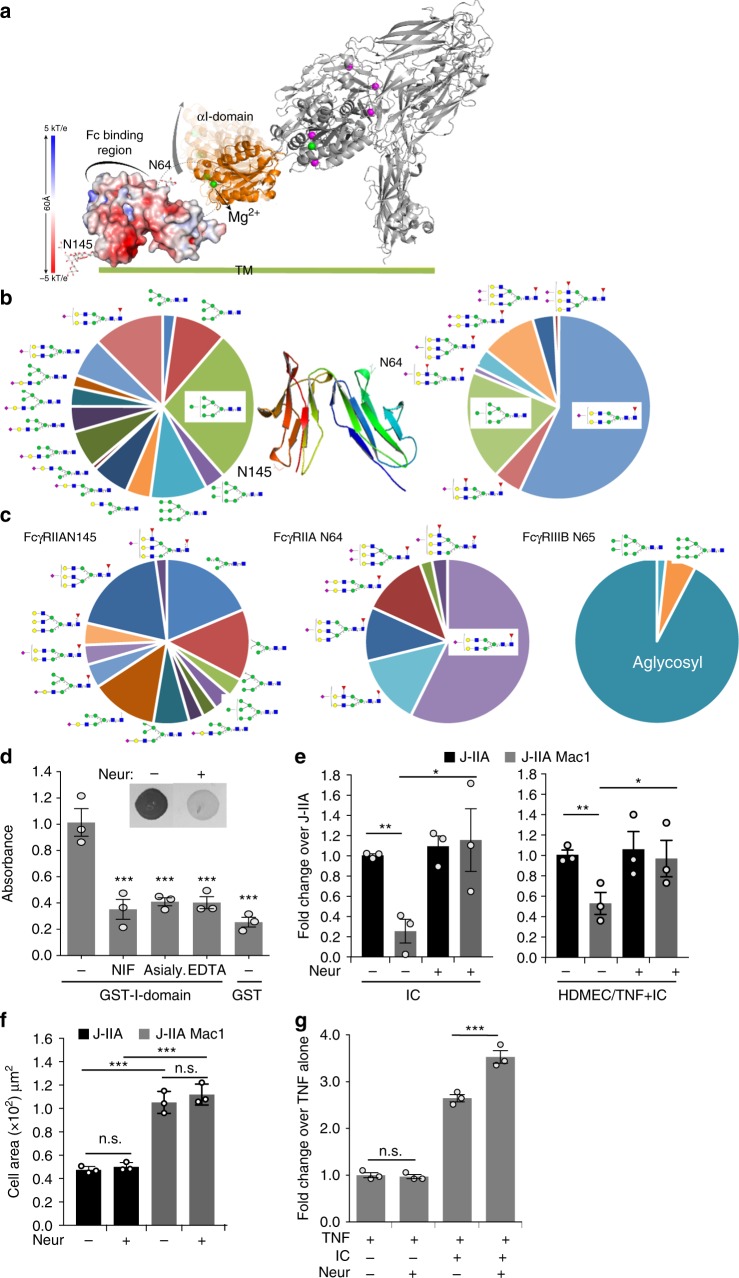
Mac-1 αI domain interaction with FcγRIIA requires FcγRIIA sialylation and divalent cations. **a** A model of glycosylated FcγRIIA and bent Mac-1 with high rotational ability and flexibility of the αI domain is depicted. Spheres are Mg^2+^ (green) and Ca^2+^ (magenta). The electrostatic surface (−5 [red] to + 5 [blue] kT/e) was calculated using the Adaptive Poisson-Boltzmann Solver software plug-in for PyMOL. **b** Site-specific glycopeptide analysis of human neutrophil derived FcγRIIA. Average relative abundance for *n* = 5 donors are shown for both sites in the context of the crystal structure of the FcγRIIA-R131 variant. **c** Site-specific glycopeptide analysis of FcγRIIA-R131 variant and FcγRIIIB NA2 allele expressed in Jurkat cells. **d** Plates coated with GST-αI domain or GST were incubated with FcγRIIA or neuraminidase A treated FcγRIIA (asialylated). As indicated, EDTA was included in the buffer to chelate divalent cations. For experimental controls, GST was incubated with FcγRIIA and GST-αI domain was incubated with NIF to show αI-domain specificity. Lectin blots were performed to detect terminal α2,6 sialic acid on FcγRIIA in the absence (−) and presence ( + ) of neuraminidase (Neur). **e** J-IIA and J-IIA Mac1 cells were incubated with neuraminidase for 30 min at 37 °C. The cells were diluted 100-fold in flow buffer and drawn over IC-coated coverslips or TNF stimulated anti-endoglin (IC) coated HDMEC coverslips at 1.0 dynes/cm^2^ and the number of adherent cells were determined as in Fig. 1. Data is represented as fold change compared to J-IIA cells (-Neur) of an average of 3 experiments ± SEM; **p < 0.01 using Student’s unpaired *t*-test. **f** Indicated Jurkat cells were incubated ± neuraminidase as in **e** and seeded onto immobilized ICs for 30 min. Coverslips were fixed, permeabilized, and stained with Alexa 568 phalloidin to visualize the actin cytoskeleton. Bar graphs represent average adherent cell area. **g** Human neutrophils ± neuraminidase (Neur) were drawn across TNF-stimulated HDMEC coated with or without anti-endoglin (IC) at 1 dynes/cm^2^ and the number of adherent cells were evaluated. Data is average of 3 experiments ± SEM. Student’s unpaired *t*-test. For **c**–**e**), **p < 0.05; ***p < 0.001 using the One way ANOVA followed by Sidak’s Multiple comparison test

Given the importance of Mac-1 αI domain in binding FcγRIIA and the reports of FcγRIIA glycosylation^45^ we posited that the divalent cation in the MIDAS motif of the αI-domain, known to be essential for Mac-1 ligand binding^17^, interacts with potential negatively charged sialic acids on FcγRIIA to reduce FcγRIIA binding affinity for ICs as depicted in Fig. 5a. FcγRIIA has been shown to contain two consensus *N*-glycosylation motifs, N64 and N145 located in the first and second Ig-like extracellular domains, respectively. These two *N*-glycans are distant from the FcγR:Fc interface and neither participates directly in the interaction based on published co-crystal structures^45^. Characterization of site-specific glycosylation for FcγRIIA on neutrophils showed that the two sites have distinct glycosylation patterns. Glycosylation at N64 is characterized by sialylated bi- and tri-antennary complex type glycans. The glycans at N64 were almost entirely sialylated with <1% neutral glycans detected across 5 donors. Antennary fucose was relatively abundant at this site with ~20–30% of species containing this motif. No aglycosylated peptide was detected for this site suggesting full site occupancy. In contrast, the glycosylation at N145 was characterized by a mixture of non-sialylated high mannose type, sialylated hybrid type and sialylated biantenanry complex type glycans. The glycans at N64 are core fucosylated complex type *N*-glycans with up to 3 antennae and up to two sialic acid residues (Fig. 5b) (Supplementary Table 1). Sialic acids were found to be both α2–6 and α2–3 linked with the former being more abundant based on the susceptibility to sialidase S. In contrast, the glycans at N145 contained equivalent levels of sialylated and non-sialylated glycans. The sialylated species consisted of hybrid type structures lacking core fucosylation and biantennary core fucosylated complex type structures (Fig. 5b) (Supplementary Table 2). The sialylated structures were capped with a single *N*-acetylneuraminic acid linked either α2–6 or α2–3 to the penultimate galactose. The relative abundance of sialylated glycans ranged from 30–60% across the 5 donors. The non-sialylated *N*-glycans were high mannose glycans ranging from 5–9 mannose residues. The relative abundance of these species ranged from 40–70% across the 5 donors. This site also appears to be fully occupied based on the absence of the aglycosylated peptide. Mass spectrometry was also used to compare the glycosylation of the conserved N64 and N65 site in FcγRIIA and FcγRIIIB, respectively that was isolated from transfected Jurkat cells, JIIA and JIIIB. The N64 from FcγRIIA was highly sialylated (Fig. 5c) while those at N145 were a mixture of high mannose, hybrid and complex sialylated, as observed for FcγRIIA in human neutrophils (Fig. 5b). In contrast, the N65 site of FcγRIIIB was predominantly (>90%) unoccupied (Fig. 5c), consistent with observations in human neutrophils (Washburn et al., Manuscript in preparation). The discrepancy in occupancy of N64/N65 between the two proteins may be explained by differences in the area around these residues in the 3D protein structure. One major difference is the presence of an *N*-glycan at N45 of FcγRIIIB that is not present in FcγRIIA. Based on the crystal structures for the two proteins this glycan could significantly influence the accessibility of N65 to glycosyltransferases. Indeed, a recent study of the highly homologous FcγRIIIA using NMR and computer simulation revealed an unexpected contact between the glycan at N45 and the protein backbone around N65^52^. The lack of sialylation of N65 in FcγRIIIB, may explain the selective inhibitory effect of Mac-1 on FcγRIIA (Fig. 3a, b) but not FcγRIIIB (Fig. 3d).

To further define the molecular interaction between FcγRIIA and Mac-1 αI-domain, we evaluated binding of recombinant GST-αI-domain with FcγRIIA using a solid phase assay. Soluble, recombinant FcγRIIA bound to GST-αI-domain coated plates significantly more than to GST and the former was inhibited by pre-treatment of the GST-αI-domain with NIF (Fig. 5d), showing specificity of the interaction. EDTA treatment significantly reduced FcγRIIA binding to GST-αI-domain suggesting a requirement for divalent cations in the observed interaction (Fig. 5d). Furthermore, neuraminidase treatment of FcγRIIA, which removed all sialic acid, markedly decreased FcγRIIA-αI-domain interactions suggesting a role for sialylation of FcγRIIA in FcγRIIA-αI-domain interactions (Fig. 5d). Next, we determined whether sialylation of FcγRIIA was required for Mac-1 inhibition of FcγRIIA mediated interactions with ICs under flow. Neuraminidase treatment of J-IIA Mac1 cells reversed Mac-1 inhibition of FcγRIIA while having no effect on J-IIA binding (Fig. 5e). Neuraminidase treatment did not affect J-IIA Mac1 mediated spreading on ICs (Fig. 5f). Similarly, human neutrophil binding to TNF/AECA coated HDMECs under flow was enhanced by neuraminidase pre-treatment (Fig. 5g), while it had no effect on neutrophil binding to endothelial cells treated with TNF alone (Fig. 5g). Together, these data demonstrate that FcγRIIA-αI-domain interactions require sialylation of FcγRIIA and divalent cations and that asialylation of FcγRIIA or removal of divalent cations interferes with Mac-1 inhibition of FcγRIIA and subsequent binding of cells to ICs under low.

### CD18 limits glomerular neutrophil influx in nephritis

**Fig. 6:**
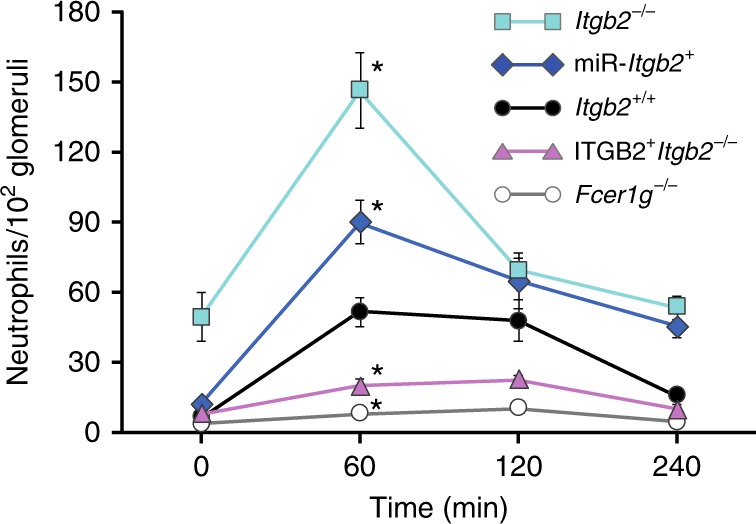
Regulation of neutrophil accumulation and injury in anti-GBM induced nephritis by CD18 integrins. Wild-type mice (*Itgb2*+/+) and mice lacking all CD18 integrins (*Itgb2*^*−/−*^), lacking all FcγRs (*Fcer1g*^*−/−*^), deficient in CD18 only in neutrophils due to neutrophil selective CD18 silencing (miR-*Itgb2* + ) or CD18 expression only in neutrophils of the CD18 deficient mice (ITGB2 + *Itgb2*^*−/−*^) were given anti-glomerular basement membrane antisera and glomerular neutrophil accumulation was evaluated at the indicated time points (*n* = 4, 4–10, 4–8, 4–6 mice of each genotype at 0, 60, 120, 240 min, respectively). Data is average ± SEM. Significant difference (*p* < 0.001) among all mouse strains was evaluated by ANOVA. *indicates mouse strains compared to *Itgb2*+/+ at 0.05 significance by Bonferroni test

Activating murine FcγRs play a key role in acute neutrophil recruitment in response to nephrotoxic (anti-GBM) antibody deposition in the glomerulus^3,4,6,53^. Recruitment is dependent specifically on FcγRIII^3^, the mouse ortholog of FcγRIIA. Here, we evaluated whether CD18 integrins regulate neutrophil recruitment via murine FcγRIII^3^, an ortholog of human FcγRIIA^2^ using genetic approaches. We observed that selective silencing of CD18 only on neutrophils using miRNA (miR-itgb2^+^)^54^ led to an increase in FcγR dependent neutrophil influx into the kidney as compared to wild-type mice (*itgb2*^*+/+*^) following anti-GBM antibody deposition despite normal peripheral blood neutrophil counts^54^. Moreover, mice lacking CD18 (*itgb2*^*−/−*^) exhibited enhanced neutrophil accumulation albeit this may reflect the peripheral blood neutrophilia observed in these mice^55^. Conversely, selective expression of CD18 on neutrophils of *itgb2*^*−/−*^ mice using the MRP8 promoter (ITGB2^+^*itgb2*^*−/−*^) led to a significant decrease in neutrophil influx as compared to CD18^*−/−*^ mice (*itgb2*^*−/−*^) (Fig. 6). Mice lacking all activating FcγRs (γ^*−/−*^) exhibited no significant neutrophil accumulation (Fig. 6), which is similar to that observed with FcγRIII mice^3^. Together, these data indicate that CD18 integrin deficiency on neutrophils increases FcγR dependent neutrophil accumulation in response to IgG/IC deposition in vivo. It is noteworthy that a deficiency in Mac-1 alone results in a significant reduction in neutrophil accumulation^25^. This is likely because LFA-1 can still dampen initial FcγRIII mediated recruitment in the absence of Mac-1 while the subsequent sustained adhesion relies on Mac-1^25,27^.

## Discussion

The dynamic regulation of immune receptors is critical for spatially and temporally tailoring the immune response. We demonstrate that the activity of FcγRIIA, a key activating FcγR on myeloid cells, is inhibited via its *cis*-ectodomain interaction with the integrin Mac-1. The inhibition is evident when rapid, high affinity interactions between FcγRIIA and IgG are required as in the case of neutrophil capture by ICs under shear stress conditions. However, once productive FcγR-IgG contacts are established, Mac-1 cooperates with FcγRIIA to promote cell spreading, a well-documented role for this integrin^19,23^. The down regulatory mechanism described herein may be particularly important in neutrophils as the inhibitory FcγRIIB (CD32b), which sets the threshold for activation of innate immune effector cells and B cells^1^ is low to absent on neutrophils of most individuals^32^.

The observed interaction between Mac-1 ligand binding αI-domain and FcγRIIA on the cell surface, and the dependence of this on divalent cations suggests that FcγRIIA is a *cis*-ligand of Mac-1. This is consistent with a previous study that reported a *cis* interaction between FcγRIIA and Mac-1 αI-domain^37^. In that case, the *cis* interaction was shown to promote cell migration albeit this only occurred when the FcγRIIA cytoplasmic tail was deleted^37^. These findings suggest the possibility that Mac-1 may exclude FcγRIIA from “signaling rich” lipid rafts, where FcγRIIA has been shown to accumulate in the absence of its cytoplasmic tail^56^, and thus reduce FcγRIIA intracellular signaling required for rapid FcγRIIA binding to complexed IgG^6^. This concept is supported by a recent finding that proteins affixed to the cortical actin cytoskeleton may serve as “pickets” that restrict FcγR lateral mobility^57^. Thus, Mac-1 could serve as a “picket”, a function that is overcome when FcγR is cross-linked by ligand and/or when Mac-1 is activated. Notably, TLR4 did not interact with the αI-domain of Mac-1 in *cis* suggesting that the ectodomain mechanism of FcγRIIA inhibition by Mac-1 does not apply for all receptors known to cooperate with Mac-1. The αI-domain of a bent integrin interacts with FcγRIIA, a *cis* ligand, as assessed by molecular modeling and FLIM, which is consistent with the emerging concept that a CD18 integrin in its bent conformation can accommodate ligand binding^43^. The lupus risk SNP, R77H is unable to inhibit FcγRIIA function or its affinity for IgG, suggesting that this SNP affects the binding affinity of Mac-1 for FcγR likely by altering allostery relay to the αI-domain needed for productive binding, as we have described for Mac-1 binding to its canonical ligand, complement C3^30^. A recent study reported a *cis* interaction between high-affinity bent Mac-1 with its known ligand, ICAM-1 that served as an anti-adhesive event^58^. Thus, *cis* interactions may more broadly serve as an important regulatory mechanism for calibrating both the activity of the integrin and in turn the heterologous receptor(s) with which it interacts.

The E^253^-R^261^ segment of Mac-1 αI-domain, which contains the binding site for ligands NIF and fibrinogen^49^, is required for Mac-1-mediated inhibition of FcγRIIA. The reversal of Mac-1’s inhibitory role by NIF suggests that the inhibition of FcγRIIA may be calibrated by the concentration and nature of *trans* Mac-1 ligands present, albeit the ability of some *trans* ligands to access the αI-domain may be hindered by a bent integrin that is in close proximity to FcγRIIA. LFA-1 can also inhibit FcγRIIA but the molecular determinants for this remain to be determined. IL-8 and sphingosine-1-phosphate can impact FcγRIIA function^5,41^. Thus, the inflammatory environment, the abundance of ICs and integrin ligands will likely dictate the relative influence of CD18 integrins on FcγRIIA mediated neutrophil recruitment following intravascular IC deposition.

FcγRIIA is glycosylated at N64 and N145 in the D1 and D2 domain respectively^45^ and deglycosylation of FcγRIII has been shown to increase FcγRIII affinity for IgG^59^. Our glycopeptide analysis of FcγRIIA isolated from neutrophils revealed that the glycans at N64 are fully processed highly sialylated structures with up to three antennae and variable levels of antennary fucose, in contrast to those at N145 which were less processed with variable sialylation. Conversely, the conserved N64 in FcγRIIIB is not sialylated, which may explain the observed inhibition of FcγRIIA but not FcγRIIIB by Mac-1. The requirement for sialylation of FcγRIIA for binding the αI-domain as determined by our solid phase assays suggests that sialic acid can bind the αI-domain. The inhibition of this interaction when divalent cations are depleted suggests that FcγRIIA-Mac-1 interaction occurs through electrostatic interactions between sialic acid and potentially negatively charged patch(es) on FcγRIIA with divalent cations in the αI-domain MIDAS motif. Interestingly, soluble *N*-acetyl glucosamine has been reported to bind the αI-domain^60^ suggesting the possibility that soluble glycans may interfere with FcγRIIA interaction with Mac-1 by competing with sialic acid-αI-domain interactions.

The importance of sialylation of the Fc region in endowing anti-inflammatory properties of IgG is now well-accepted^61,62^. In our studies, we demonstrate that FcγR sialylation has down regulatory properties, which indicates a new anti-inflammatory function for sialylation in immune responses. The specific mechanism by which the αI -domain may interfere with FcγRIIA is not clear but may involve physical interference of the Fc binding region of FcγR by Mac-1, given the significant difference in size of Mac-1 relative to FcγRIIA. It is also possible that Mac-1 interaction with FcγRIIA glycans in the Ig-like domain(s) hinders the reported 10° shift of the D2 domain that occurs upon Fc-binding^63^, prevents FcγRIIA dimerization^45^, and/or excludes FcγRIIA from lipid rafts^56^.

We show that FcγRIIA inhibition by Mac-1 is physiologically relevant. Neutrophils from patients lacking CD18 integrins and normal neutrophils treated with neuraminidase exhibited enhanced FcγRIIA mediated recruitment to IgG deposited on cytokine-activated endothelium in vitro compared to untreated neutrophils. Furthermore, in mice, CD18 integrin deficiency specifically in neutrophils exhibited enhanced glomerular neutrophil accumulation in an acute model of FcγR dependent anti-GBM nephritis while expression of human CD18 integrin specifically on neutrophils reversed the enhanced neutrophil influx observed in mice with global CD18 deficiency.

In conclusion, our study revealed an ectodomain interaction between two major adhesion receptors, FcγRIIA and Mac-1 that modulates neutrophil recruitment to deposited IgG, thus providing a mechanism by which the activity of FcγRIIA is regulated in autoimmune mediated neutrophil recruitment. This method of regulation by Mac-1 differs from the intracellular mechanisms described for its regulation of heterologous receptors^19^. Our data also reveals that the interaction of sialylated FcγRIIA with Mac-1 down-regulates FcγRIIA activity, which suggests that cell-type specific sialylation of FcγRIIA on myeloid cells may contribute to diversity in FcγRIIA mediated responses. The calibration of FcγRIIA mediated neutrophil responses to vascular deposited ICs facilitated by *cis* interactions with Mac-1, leads us to predict that a similar interaction may affect other FcγRIIA functions that require short duration, high affinity interactions with complexed IgG in myeloid cells expressing these two opsonic receptors (e.g. capture and phagocytosis of live microbes). Finally, our studies suggest that therapeutic agents that maintain Mac-1 allosterically in a bent state may be able to control the deleterious effects of FcγRIIA mediated neutrophil accumulation in autoimmune disease.

## Methods

### Mice

Wild-type mice (*Itgb2* + / + ) and mice lacking all CD18 integrins (*Itgb2*^*−/−*^)^55^, lacking all FcγRs (Fcer1g^*−/−*^)^64^, deficient in CD18 only in neutrophils due to neutrophil selective CD18 silencing (miR-itgb + ) or CD18 expression only in neutrophils of the CD18 deficient mice (ITGB2^+^itgb2^*−/−*^)^42^ were maintained in a specific pathogen-free facility at the New Research Building animal housing facility at Harvard Medical School. Mice used for each experiment were 7 weeks of age and were age and sex matched. The Harvard Medical School Animal Care and Use Committee approved all procedures in this study.

### Human neutrophils

Human polymorphonuclear neutrophils (PMNs;>85% pure) were isolated from whole blood drawn from healthy volunteers. Blood was drawn and handled according to protocols for protection of human participants approved by the Brigham and Women’s Hospital Institutional Review Board and National Institutes of Allergy and Infectious Diseases, and all volunteer participants gave written informed consent. Briefly, anticoagulated whole blood (sodium citrate 1:9, pH 6.5) was collected by venipuncture from normal donors and diluted 1:1 with Hanks’ balanced salt solution (HBSS) without calcium and magnesium. PMNs were separated by centrifuging on a histopaque (Sigma) separation medium followed by dextran (1%) sedimentation of RBCs. The isolated cells were maintained at 8 °C and used immediately. De-identified LADI patient blood samples drawn at National Institute of Allergy and Infectious Diseases, Laboratory of Clinical Infectious Diseases, National Institutes of Health and age and sex matched normal, healthy controls, were collected in ACD anticoagulant in BD vacutainer tubes and shipped overnight at room temperature. Upon receipt, PMNs were purified from whole blood using the Easysep direct human neutrophil isolation kit from Stemcell Technologies as per manufacturer instructions.

### Reagents and antibodies

Human IgM (Jackson ImmunoResearch), Protein A (Pierce Thermo Scientific), Human ICAM-1 Fc (R&D), Neutrophil inhibitory factor (NIF) (R&D), Fibronectin (Sigma-Aldrich), TNFα (Peprotech), and fMLP (Sigma-Aldrich) were purchased. Serum used as a source of iC3b was obtained from healthy volunteers. Blood was drawn and handled according to protocols for protection of human participants approved by the Brigham and Women’s Hospital Institutional Review Board, and all volunteer participants gave written informed consent. Anti-human CD11b clone ICRF44 (Biolegend; cat#301310), anti-human CD11b clone M1/70 (Biolegend; cat#101216) and rat IgG2a isotype control (eBioscience; cat#14-4321-82), anti-human CD32a clone IV.3 (Stemcell Technologies; cat#60012) and secondary antibodies anti-mouse IgG AF488 (Invitrogen; cat#A28175) and anti-mouse IgG AF594 (Invitrogen; cat#R37121) were purchased as indicated and used at 1:100-1:1000. Anti-endoglin/CD105 (Biolegend; cat#323202) and rabbit anti-mouse (Dako; cat#Z0259) antibody were used at 1:250 and 1:500 respectively.

### Lentiviral constructs and Jurkat cells

The R77H mutation was introduced into the human CD11b using standard PCR approaches. The E^253^–R^261^ mutation was introduced by replacing CD11b’s E^253^–R^261^ EDVIPEADR with SGNIDAAKD present in CD11a^49^ using an overlap PCR strategy with the following primers: sense 5′-AGCGGCAACATCGATGCTGCAAAAGACGAGGGAGTCATTCGCTACG-3′ and antisense 5′-GTCTTTTGCAGCATCGATGTTGCCGCTATATCCCAAGGGATCGC-3′ and the mutation was confirmed by DNA sequencing. Replacement of the NIF binding E^253^-R^261^ domain of Mac-1 with the homologous domain of LFA-1 is likely disruptive because this region, which corresponds to the α5-helix, is not equal in length in Mac-1 and LFA-1. The Mac-1 domain has an additional five residues, which structurally adopts an extra 2.5 helical turn. Replacing this region with the CD11a sequence likely causes a structural change not only in this region but also to peripheral segments on the αI-domain that impairs Mac-1’s affinity for FcγRIIA. cDNA constructs for CD11b^WT^, CD11b^R77H^, CD11b^E253-R261^, FcγRIIA (CD32a), or FcγRIIIB (CD16b) were cloned in the lentiviral plasmid pWPI (modified from the Addgene, plasmid #12254, from Didier Trono laboratory, by removing the EGFP cassette). 293 T cells (clone 17, from ATCC) were transfected with the lentiviral construct (pWPI) in addition to the packaging plasmids psPAX2 and pMD2.G (Addgene, plasmids #12260 and #12259 from the laboratory of Didier Trono) using Lipofectamin (Invitrogen). Supernatant of transfected cells were passed through a 0.45 μm filter and used to transduce wild-type Jurkat cells or Jurkat cells lacking CD11a^34^. All cell lines were sterilely sorted on a BD FACS Aria to obtain populations of cells with similar levels of desired proteins on their surface. Clones were cultured in RPMI 1640 supplemented with 10% FCS, 2 mM of l-glutamine and penicillin/streptomycin (0.1 mg/ml) (Lonza) and used in functional assays.

### Adhesion assay under shear stress

For binding to immune complexes, glass coverslips were incubated with preformed soluble BSA-anti-BSA immune complexes (ICs)^3^ in a circumscribed 5 mm circle within the 25 mm diameter glass coverslips for 2 h at room temperature. The coverslips were mounted on a parallel plate flow chamber and maintained at 37 °C. Cells were perfused in RPMI 0.1% BSA at indicated shear stress. For binding to human dermal microvsacular endothelial cells (Lonza), the endothelial cells were grown on coverslips, activated with human TNFα (10 ng/mL) for 4 h and incubated with a mouse anti-human endoglin mAb (anti-hCD105) and subsequently with rabbit anti-mouse IgG for 15 min at 37 °C. For experiments with NIF, αI-domain or neuraminidase, Jurkat cells were pretreated with NIF (10 nM), αI-domain (100μg/ml), or neuraminidase (500U/ml) for 30 min at 37 °C. Cells (1 × 10^6^/ml) were perfused over endothelial cells at 1.0 dyne/cm^2^. After 2 min of continuous flow to allow the cells to equilibrate, the number of adherent cells in four random fields was visualized for 10 s for each coverslip. Two coverslips (total 8 fields) were recorded for each condition and the number of cells per field was averaged. Live imaging of cell adhesion was recorded on a Nikon a TE2000 inverted microscope (equipped with a 20 × /0.75 NA phase contrast objective) coupled to a video camera. VideoLab software (Mitov, Moorpark, CA) was used for the recording.

### Analysis of cell spreading under static conditions

Cells were seeded onto IC-coated coverslips for 30 min. Coverslips were fixed with 4% PFA, permeabilized and stained with Alexa 568 phalloidin to visualize the actin cytoskeleton. Adherent cell area was calculated on ImageJ (NIH).

### Biomembrane force probe assays

Human red blood cells (RBCs) were isolated from whole blood of healthy volunteers by finger prick according to protocols approved by the Institutional Review Board of Georgia Institute of Technology. Freshly isolated human RBCs were biotinylated and glass beads were coated with streptavidin (SA) plus hIgG1, Mac-1, or FcγRIIA as previously described^65^. A coated bead was attached to the apex of a micropipette-aspirated biotinylated RBC, which together acted as an ultrasensitive force transducer. The aspiration pressure was set to control the probe stiffness at 0.2 or 0.3 pN/nm. The axial displacement of the bead-RBC interface was monitored by high-speed camera, which reflected the force on the RBC by Hooke’s law. Jurkat cells expressing FcγRIIA and/or Mac-1 were aspirated by an opposing micropipette. The Jurkat cell was programmed to repeatedly approach and contact the probe bead for defined contact times (*t*_c_, 0.1–5 s) to allow bond formation, which was signified by a tensile force on the RBC upon retraction of the cell. The approach-contact-retraction cycle was repeated 50 times for at least 3 cell-bead pairs at each *t*_c_ to calculate a mean ± SEM of adhesion frequency, *P*_a_. Then the 2-dimensional (2D) effective affinity (*A*_c_*K*_a_) and off-rate (*k*_off_) were derived by fitting the *P*_a_ vs *t*_c_ curve to a 2D kinetics model^66^ with the following equation:

$$P_{\rm{a}} = 1 - {\rm{exp}}\left\{ { - m_{\rm{r}}m_{\rm{l}}A_{\rm{c}}K_{\rm{a}}\left[ {1 - {\rm{exp}}\left( { - k_{{\rm{off}}}t_{\rm{c}}} \right)} \right]} \right\}$$

where *m*_r_ and *m*_l_ are the respective site densities of receptor (FcγRIIA or Mac-1) and ligand (hIgG1, Mac-1, or FcγRIIA).

The site densities of hIgG, Mac-1, or FcγRIIA on the probe beads, and the site densities of FcγRIIA and/or Mac-1 on the Jurkat cells were measured by antibody immunostaining and flow cytometry^65^. As a confirmation of the binding specificity, beads coated with SA alone contacting the same batch of Jurkat cells only rendered ~3% adhesion frequency. For experiments with NIF or αI-domain, Jurkat cells were pretreated with NIF (10 nM) or I-domain (100 μg/ml) for 30 min at 37 °C before being positioned onto the opposing micropipette.

### FLIM analysis

Jurkat cells or human neutrophils were stained with purified anti-human CD11b (ICRF44 or M1/70) followed by anti mIgG AF594. Cells were washed and stained with FITC labelled anti-human FcγRIIA. Cells were washed, fixed and immobilized onto poly-l-Lysine coated glass coverslips and mounted in Vectashield (Invitrogen). Protein interactions were defined by time-correlated single-photon counting FLIM as previously described^67^. The fluorescence baseline lifetime of FITC (donor fluorophore, FcγRIIA) was calculated by single-exponential-decay fitting of fluorescence emission in the absence of Alexa Fluor 594 (acceptor fluorophore). For samples stained for both donor and acceptor, lifetimes were fitted to a biexponential decay with lifetime of one component fixed to the donor-only lifetime. Three separate experiments were performed, with fluorescence lifetime (tm) reported as an average of total number of cells analyzed, and within each cell at least 10 different areas were used to determine the mean value. A Plan APO VC 60 × oil DC N2 objective 1.4 NA, mounted on a Nikon Ti-E inverted microscope equipped with filter cubes used for DAPI, fluorescein isothiocyanate, and tetramethylrhodamine isothiocyanate fluorophores (Nikon), was used for epifluorescence and FLIM as described^67^. Nikon Elements 3.10 imaging software was used to collect epifluorescence data. For FLIM acquisition, Becker and Hickl (Berlin, Germany) a BDL-488-SMC Picosecond Diode Laser and both long-pass (HQ500LP) and bandpass (HQ435/50) emission filters were used in combination with a hybrid detector (HPM-100-40 GaAsP hybrid detector) integrated with a Hamamatsu (Hamamatsu, Japan) R10467-40 hybrid photomultiplier tube. Becker and Hickl SPCM software with detector controller card was used to acquire FLIM data, and SPCImage 3.0 (Becker and Hickl, Berlin, Germany) software was used for FLIM analysis.

### Solid phase binding assay

96 well Nunc plates (Thermofisher Scientific) were coated with GST αI-domain (20 µg/mL) or GST, followed by blocking with 2% BSA solution for 1 h. After washing with TBST, the buffer plate was incubated for 3 h at room temperature in the presence of FcγRIIA (40 µg/mL) or asialylated FcγRIIA (40 µg/mL), NIF (20 nM) followed by FcγRIIA in TBS-Tween and FcγRIIA with 2 mM EDTA. After each step, the plate was washed with TBST and bound FcγRIIA was detected using mouse anti-human CD32 antibody (clone IV.3, Stemcell Technologies) followed by anti-mouse HRP conjugated antibody. The plates were developed with 3,3,5,5-tetramethylbenzidine (TMB) HRP substrate, the reactions were quenched using 2 M sulfuric acid and absorbance was measured at 450 nm.

### Enzymatic removal of FcγRIIA-sialic acid and lectin blots

Recombinant FcγRIIA (CD32a) (generated in HEK293 cells, Acros Biosystems) was incubated overnight with 500 U/ml α2–3,6,8 Neuraminidase (New England Biolabs) to cleave sialic acid. Recombinant FcγRIIA expressed in HEK293 cells is glycosylated^68^. A lectin blot was used to confirm asialylation^69^. FcγRIIA and asialylated FcγRIIA was blotted on nitrocellulose membrane followed by blocking with 2% BSA, and the membrane was probed with biotinylated sambucus nigra lectin (SNA 5 μg/mL, Vector Laboratories) to detect terminal α2,6 sialic acid.

### Characterization of human FcγRIIA and FcγRIIIB glycosylation

Neutrophil FcγRIIA was isolated from ~5 million neutrophils from blood drawn from 5 healthy donors as part of the Momenta Pharmaceuticals blood donor program. The collection, handling and analysis of healthy human neutrophils, per experimental protocol 102013-001, were approved by the Western Institutional Review Board. Neutrophils were isolated from lysed whole blood through negative selection using the Neutrophil Enrichment Kit (Stemcell Technologies). Freshly isolated neutrophils were pelleted and frozen at −80 °C until they were used for analysis. Likewise, FcγRs were isolated from 20 million Jurkat cells expressing either the FcγRIIA R131 variant or FcγRIIIB NA2 allele. Proteins were immunoprecipitated using biotinylated goat polyclonal antibodies against human FcγRII (R&D Systems) or human FcγRIII (R&D Systems) The proteins were isolated from cells by first spinning down cells at 300 g for 2 min and then washing 3 × 500 μL of ice cold PBS. Then 75 μL of IP Lysis Buffer (ThermoFisher Scientific) was added to each sample and cells were lysed by sonication and cell debris spun out at 10,000 g for 5 min. FcγRs were immunoprecipitated with 2 μg of biotinylated goat polyclonal antibodies. The antibody-FcγR complex was isolated using streptavidin magnetic beads (ThermoFisher Scientific). The beads were washed two times with 500 μL of IP Lysis buffer and two times with 500 μL of ice cold PBS. The bound protein was eluted by incubating the beads in 50 μL of 6 M guanidine HCl for 30 min at 65 °C. The eluted protein was reduced for 30 min at 65 °C with DTT at a concentration of 25 mM. Free cysteine residues were alkylated with iodoacetamide at a concentration of 75 mM. The isolated proteins were dialyzed across a 10 KDa membrane against 4 L of 25 mM ammonium bicarbonate for 18 h at 4 °C prior to proteolysis. The immunoprecipitated proteins were digested with chymotrypsin for 4 h at room temperature. Glycan pictures were generated using GlycoWorkbench (Fig. 5b, c). Glycopeptides from FcγRIIA were initially identified using a top 12 data dependent acquisition and searching the MS/MS scans for the characteristic Y1 ion (Supplementary Figure 4) for each of the predicted chymotryptic glycopeptides (Supplementary Table 1). Masses with spectra generating these characteristic fragments along were included in a targeted MS/MS method (Supplementary Table 2) for characterization of neutrophil FcγRIIA. Fragmentation of glycopeptides from both sites yielded abundant specific Y1 ions (Supplementary Figure 5). Quantitation of the targeted glycopeptides was based on the extracted ion area of Y1 fragment (Supplementary Figure 6 and 7). FcγRIIIB quantitation of the N65 glycosylation was based on the extracted ion area of the doubly charged precursor for the aglycosylated chymotryptic peptide and the triply charged precursor ions for glycosylated peptides.

### Acute anti-glomerular basement membrane nephritis

7-week-old male mice were given an intravenous injection of 300 μL of nephrotoxic serum without prior preimmunization. Mice were euthanized after 60, 120, 240 min of injection and both kidneys were harvested for histological evaluation. Neutrophils were identified by the chloroacetate esterase reaction as reported^3^. For each animal, glomerular neutrophil counts in more than 100 glomeruli per kidney section were made.

### Statistical analysis

All data obtained are presented as the mean ± SEM. Statistical differences were analyzed with a 2-tailed unpaired *t*-test, or one way ANOVA followed by Sidak’s or Bonferoni’s multiple comparison test as indicated. *P* values <0.05 were considered significant.

## Electronic supplementary material

Supplementary Information

## Data Availability

All relevant data are included in the paper and/or its supplementary information files.
