# Supplementary material for: Cis interaction between sialylated FcγRIIA and the αI-domain of Mac-1 limits antibody-mediated neutrophil recruitment
Source: Nat Commun. 2018 Nov 29;9:5058. doi: 10.1038/s41467-018-07506-1 (PMC6265255; doi:10.1038/s41467-018-07506-1)
Supplement: Supplementary file 1 — Supplementary Information [file 41467_2018_7506_MOESM1_ESM.pdf]

File name: Supplementary Information
Description: Supplementary Figures

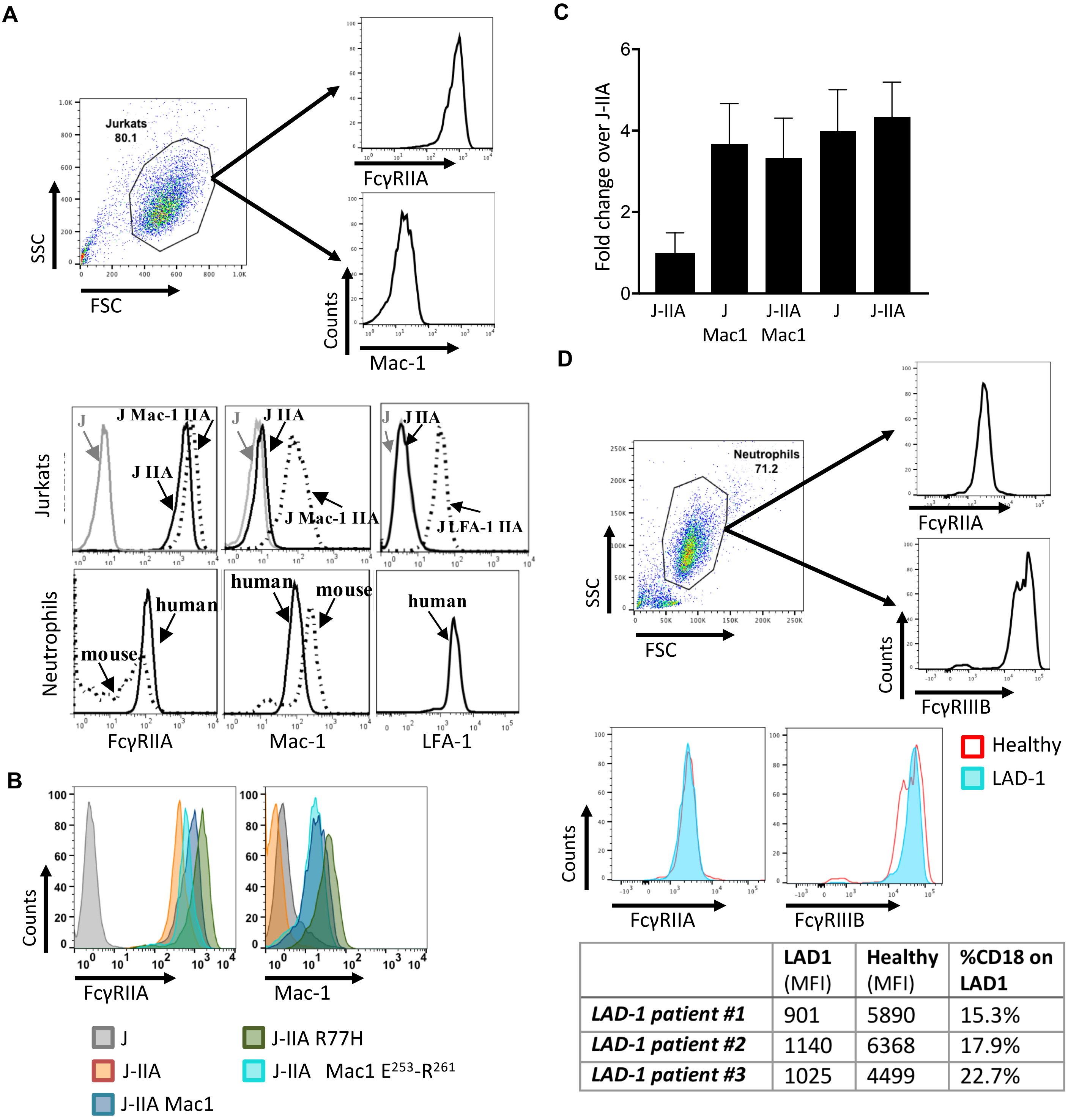

	LAD1 (MFI)	Healthy (MFI)	%CD18 on LAD1
LAD-1 patient #1	901	5890	15.3%
LAD-1 patient #2	1140	6368	17.9%
LAD-1 patient #3	1025	4499	22.7%

Supplementary Figure 1: Flow cytometric analysis of FcγR and Mac-1 expression. A) Characterization of J-IIA±Mac-1 and J-IIA±LFA-1. Top panel shows gating strategy for Jurkats. Bottom panel shows expression of each receptor on jurkat cells and comparison with human and mouse neutrophils from mice expressing human FcγRIIA. *(Gating strategy for purified neutrophils is shown in D).* **B)** Comparison of FcγRIIA and Mac1 expression on indicated Jurkat cell lines. Same gating strategy as in A was followed. **C)** Cells were perfused under flow conditions over TNF activated human dermal microvascular endothelial cells (HDMEC) without ICs. The number of adherent cells was assessed and averaged. Bar graph represents fold change calculated over J-IIA. n=1 with duplicate coverslips per condition. **D)** Top panel shows gating strategy for purified human neutrophils. Bottom panel shows characterization of FcγRIIA, FcγRIIB and CD18 surface levels on healthy vs LAD-1 patient neutrophils. A representative image of FcγR expression and the mean fluorescence intensity (MFI) of CD18 on 3 normal and 3 LAD1 patients is shown. The %CD18 on LAD1 as compared to healthy (100%) is calculated.

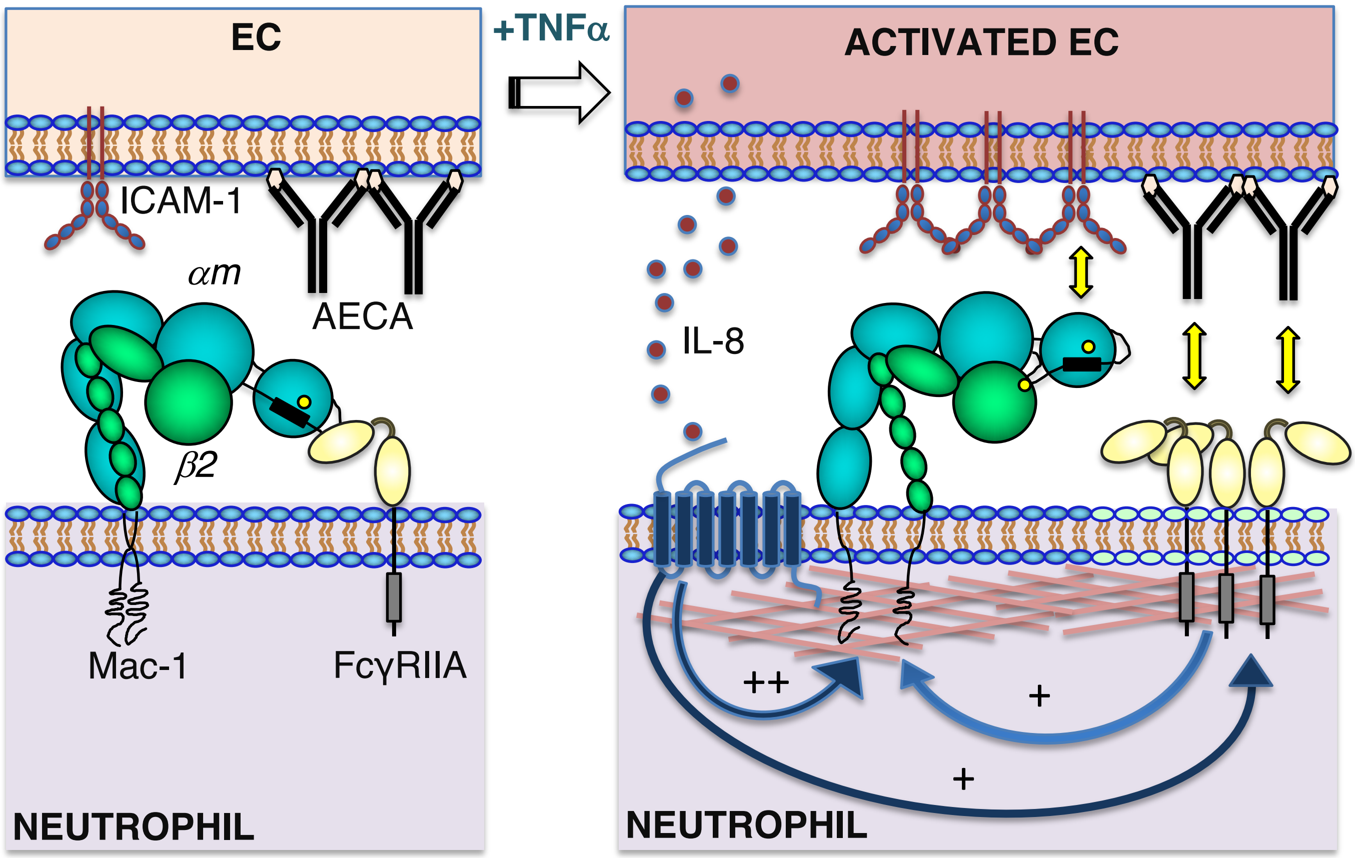

Supplementary Figure 2: Model of CD18 regulation of FcγRIIA mediated neutrophil recruitment to AECA-coated endothelial cells based on results In Figure 1 and published data. In resting neutrophils, Mac-1 I-domain interacts in *cis* with sialylated FcγRIIA and therefore prevents FcγRIIA interaction with AECAs. TNF stimulation of the endothelium results in the generation of a chemokine, such as IL-8 which interacts with its GPCR to activate Mac-1 via inside-out signaling (++), which puts the integrin in a high affinity state for ICAM-1 binding. The interaction with ICAM-1 induces outside-in signaling for further integrin activation and neutrophil adhesion. Both inside-out and outside-in signaling are required for optimal and stable integrin activation. Integrin activation disrupts Mac-1 interactions with FcγR thus allowing FcγR binding to AECA. GPCR stimulation also enhances FcγRIIA function potentially by facilitating actin polymerization, distribution to lipid rafts and clustering, known to enhance FcγR activity.

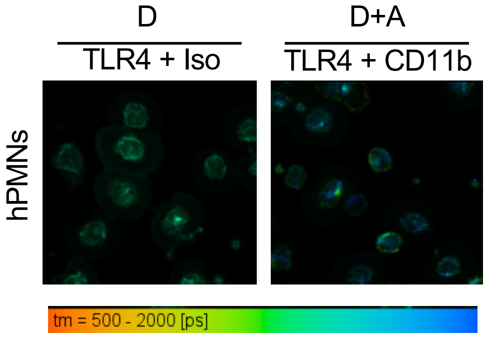

	FcγRIIA Fluorescence lifetime tm (ps)	
**Antibodies**	**TLR4 + Iso**	**TLR4 + CD11b**
**hPMNs**	1694 ± 112.65	1784 ± 201.89

Supplementary Figure 3: Analysis of molecular interactions between Mac-1 and TLR4. FLIM was conducted on human neutrophils expressing Mac-1 and TLR4 by staining with the donor (D) antibody for anti-TLR4 (AF488) and/or the acceptor (A) antibody for Mac-1 (ICRF44 AF568). FLIM images were taken and presented in pseudocolors from red to green. The fluorescence lifetimes of Alexa Flour 488 in the absence (D) or presence (A+D) of the acceptor Alexa Flour 568 antibody were calculated. Data is Average±SD.

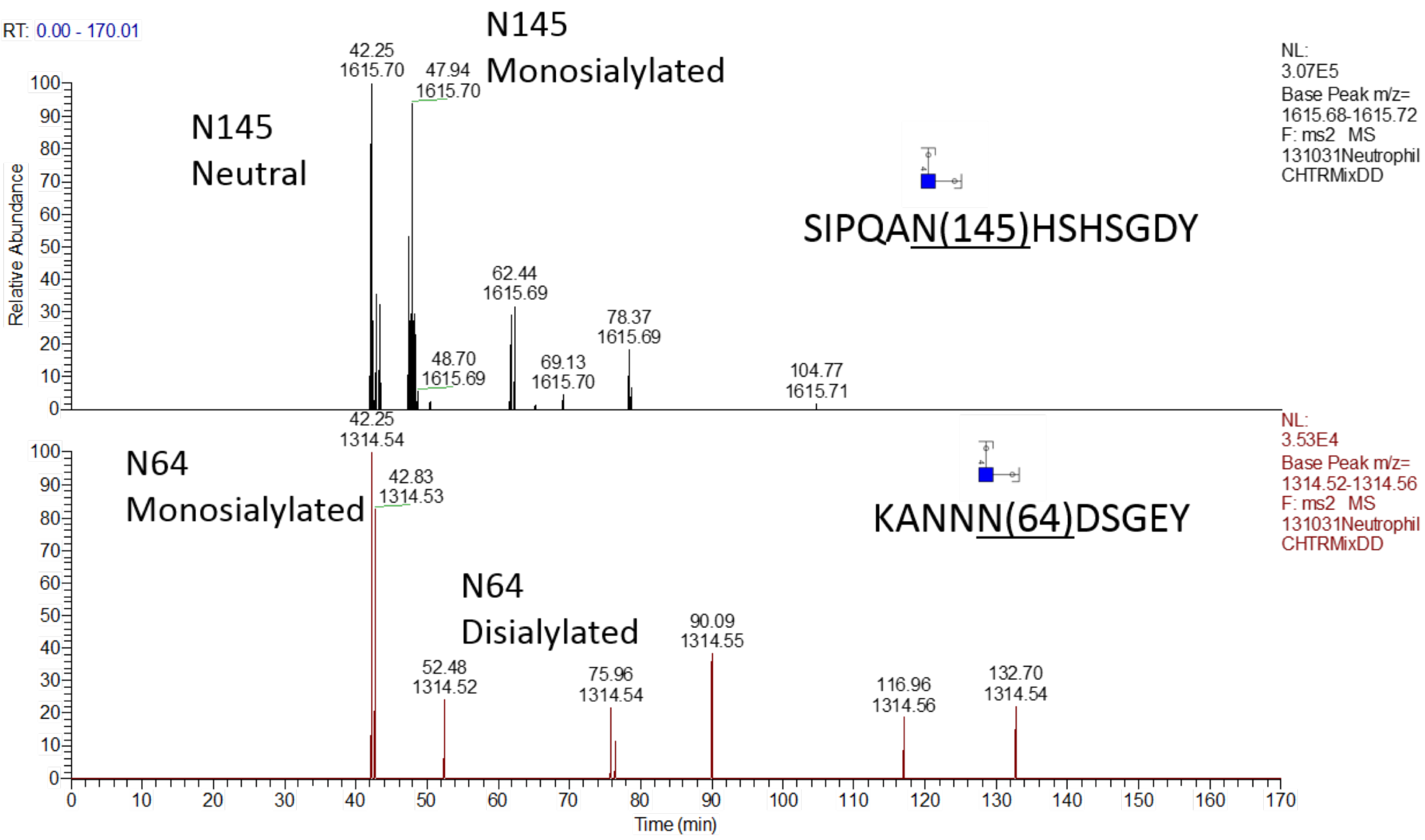

Supplementary Figure 4. Y1 fragment scanning from the MS/MS for FcγRIIA glycopeptides from the data dependent acquisition showing the elution pattern of the neutral, monosialylated and disialylated glycans from N145 (top panel) and N64 (bottom panel)

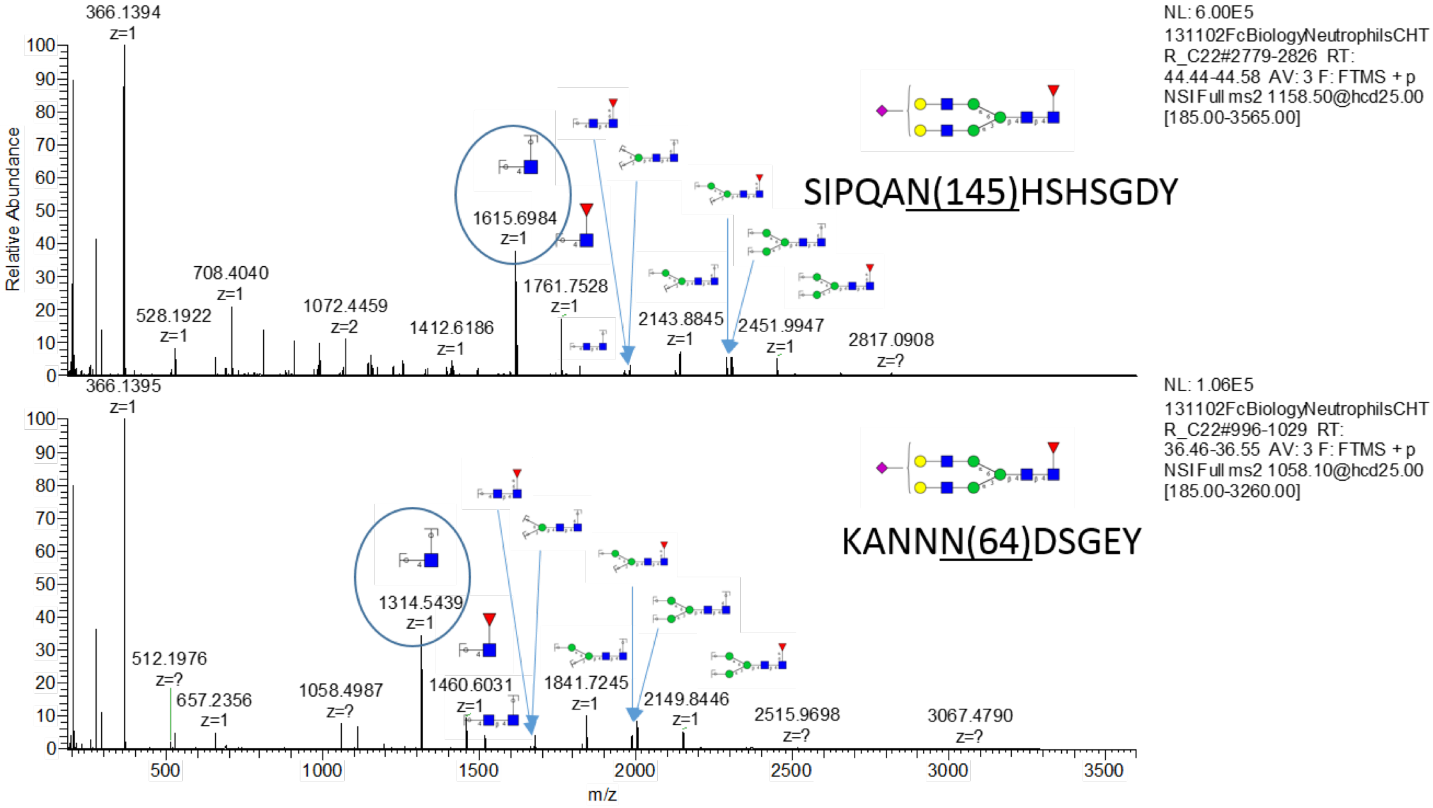

Supplementary Figure 5. Annotated MS/MS spectra for FA2G2S1 for N145 (top) and N64 (bottom) showing the fragmentation pattern for the two glycopeptides with the Y1 ion used for quantitation circled.

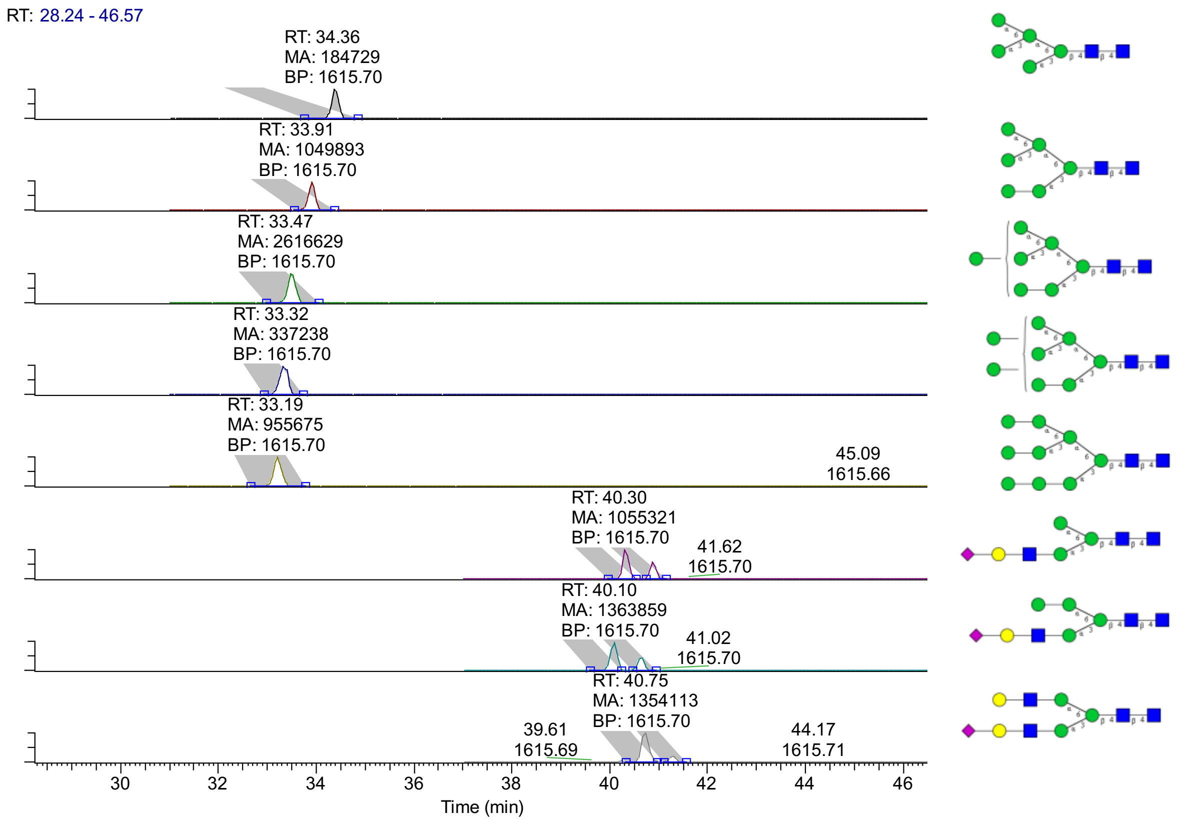

Supplementary Figure 6 Y1 Extracted ion chromatogram for N145 glycopeptides showing the elution pattern of neutral and sialylated glycopeptides on the data independent acquisition used for relative quantitation of the N145 glycopeptides.

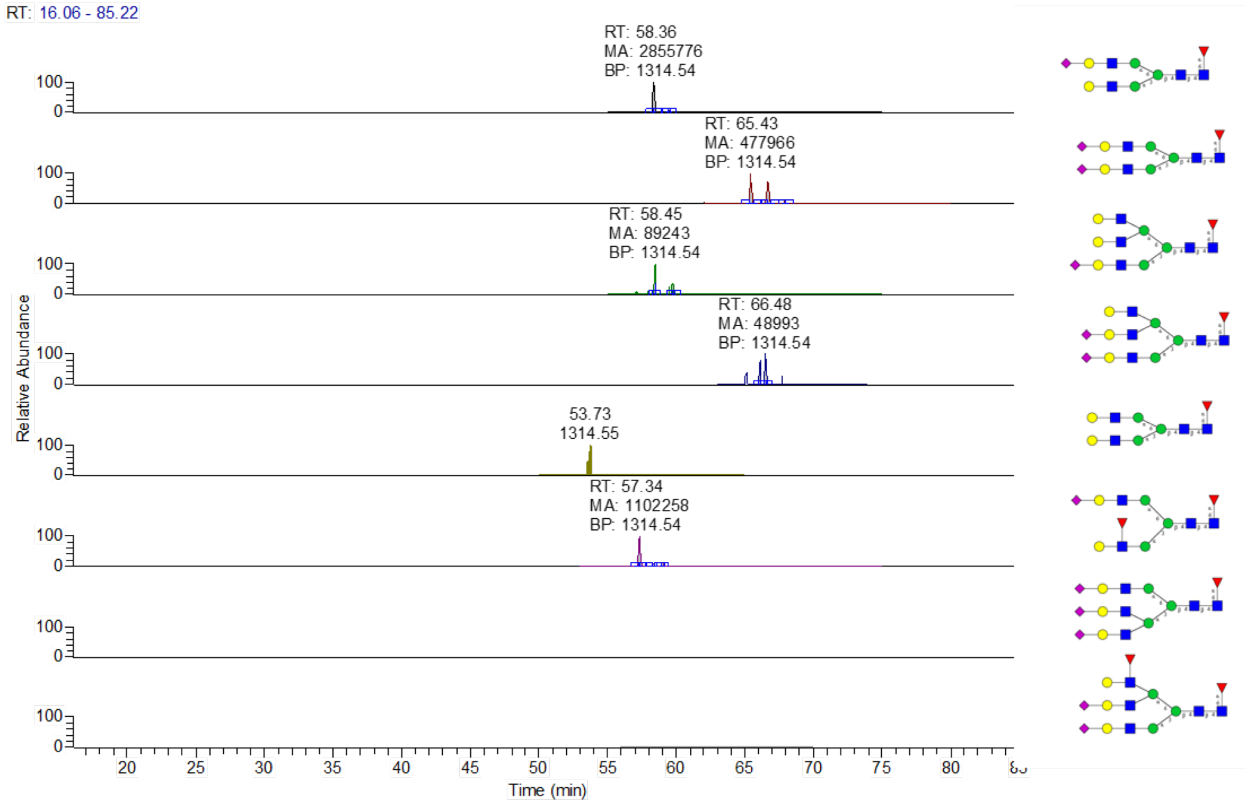

Supplementary Figure 7. Y1 Extracted ion chromatogram for N64 glycopeptides showing the elution pattern of neutral and sialylated glycopeptides on the data independent acquisition used for relative quantitation of the N64 glycopeptides.

Sequence	Site	Y1 m/z (z=1)
**KANNN(64)DSGEY**	FcγRIIA N64	1314.54
**SIPQAN(145)HSHSGDY**	FcγRIIA N145	1615.70

Supplementary Table 1. Peptide sequence for chymotryptic FcγRIIA glycopeptides and resulting Y1 ion mass
